# Adaptive Tracking Control for the Piezoelectric Actuated Stage Using the Krasnosel’skii-Pokrovskii Operator

**DOI:** 10.3390/mi11050537

**Published:** 2020-05-25

**Authors:** Rui Xu, Dapeng Tian, Zhongshi Wang

**Affiliations:** 1 Changchun Institute of Optics, Fine Mechanics and Physics, Chinese Academy of Sciences, Changchun 130033, China; xur@ciomp.ac.cn (R.X.); zhongshiwang@ciomp.ac.cn (Z.W.); 2 Key Laboratory of Airborne Optical Imaging and Measurement, Chinese Academy of Sciences, Changchun 130033, China; 3 Research Institute of Intelligent Control and Systems, Harbin Institute of Technology, Harbin 150001, China

**Keywords:** adaptive control, hysteresis, Krasnosel’skii-Pokrovskii operator, piezoelectric actuated stage

## Abstract

In this paper, a discrete second order linear equation with the Krasnosel’skii-Pokrovskii (KP) operator is used to describe the piezoelectric actuated stage. The weights of the KP operators are identified by the gradient descent algorithm. To suppress the hysteresis nonlinearity of the piezoelectric actuated stage, this paper proposes an adaptive tracking control with the hysteresis decomposition on the designed error surface. The proposed adaptive tracking controller dispenses with any form of the feed-forward hysteresis compensation and the unknown parameters of the discrete second order linear equation are adaptively adjusted. Some simulations are implemented to verify the effectiveness of the KP operators, then a series of modeling and control experiments are carried out on the piezoelectric actuated stages experimental systems. The comparative experimental results verify the feasibility of the KP operators modeling method and the adaptive tracking control method.

## 1. Introduction

Piezoelectric actuated stages are extensively applied in the high-precision imaging mechanism and ultra-precision positioning field, such as optical alignments, scanning tunneling microscope, and photoelectric imaging tracking systems, because of their high response speed, big work-distance and strong stress [1,2]. However, the hysteresis of the piezoelectric materials damages the positioning accuracy of the stage [3]. To solve this problem, numerous modeling methods have been proposed to capture hysteresis of the piezoelectric actuated stage, which mainly include the differential equations-based models and operator-based models. So far, the Duhem model, Bouc-Wen model, and Coleman-Hodgdon model are the common differential equations-based models [4]. For example, the Coleman-Hodgdon model, which had two differential equations with different exponential, was used to describe the rising and declining hysteresis curves [5]. Lin et al. [6] conducted a depth research for a class of generalized Duhem models. Experimental results demonstrated that the generalized Duhem model can describe the hysteretic characteristics more accurately. In [7], the Bouc-Wen model was used to represent the hysteresis nonlinearity of the piezoelectric actuated stage and it got the great results. The Preisach model describes the hysteresis nonlinearity by the weighted summation of series of Preisach operators with the values of 0–1 [8,9,10]. The Prandtl-Ishinskii (PI) model is a modification form of Preisach model [11,12]. The PI model exploits two basic operators of Play and Stop [13]. Furthermore, it obtains widespread application for its simple mathematical structure. For example, Kuhnen K et al. [14] investigated a modified PI model in which the different operators have different thresholds. The Krasnosel’skii-Pokrovskii (KP) model is another modification form of the Preisach model [15]. The element operator of KP model overcomes the drawback of the jump discontinuities of Preisach operators. Moreover, it includes minor loops within its major loop which make it more closed to the actual behaviors of the hysteresis nonlinearity [16,17]. In [18], a rate-dependent KP model is proposed to describe the dynamic hysteresis nonlinearity of the piezoelectric-driven platform. The unknown parameters are identified by the interior point method. The simulation results show the proposed hysteresis model can describe the hysteresis loop with the different frequencies driving input of the piezoelectric-driven platform.

Based on above hysteresis models, some control methods have been designed to eliminate the hysteresis effects and achieve high-precision trajectory tracking control for the piezoelectric actuated stage, such as the inverse feed-forward control [19], sliding mode control [20,21,22], and disturbance rejection control [23]. Merry et al. [24] designed a feedforward compensation controller based on an extended Coleman-Hodgdon model to eliminate the hysteresis nonlinearity. Li et al. [25] proposed an inverse compensation control method with the inverse rate-dependent PI model to solve the hysteresis of the piezoelectric actuated platform and achieve high-precision positioning control. Nguyen et al. [26] utilized a discrete-time quasi-sliding-mode control with a new sliding variable to alleviate the effects of the hysteresis nonlinearity. Shan et al. [27] proposed a robust output feedback controller to degrade the hysteresis nonlinearity of piezoelectric actuated systems, the tracking error remained less than 5%. Cao et al. [28] presented a hybrid control method by integrating the inversion feedforward control and PID-based sliding model control to compensate the hysteresis nonlinearity more effectively. Ge et al. [29] proposed a hybrid controller comprised of inverse Preisach model as feedforward compensation and PID control to improve the tracking accuracy for a piezoelectric actuated system, when the amplitude of reference displacement is 12 μm, the maximum error rate is 2.08%. While Lin et al. [30] developed an intelligent integral backstepping sliding-mode controller using a recurrent neural network to eliminate the hysteresis nonlinearity of the piezo-driven nanopositioning stage. Xu [31] utilized the dynamical model of the piezo-stage to design a digital integral terminal sliding mode controller, the effectiveness of which was supported with experimental results.

This study adopts a discrete second order linear equation with hysteresis input to express the piezoelectric actuated stage. Next, an adaptive tracking controller based on the new designed error surface is proposed to mitigate the hysteresis nonlinearity of the piezoelectric actuated stage, where the hysteresis model of this stage is separated on the proposed error surface to facilitate the design of the adaptive control approach. Comparative experimental results show that the output displacement of the piezoelectric actuated stage with the proposed adaptive controller can track the desired trajectory signal, its hysteresis characteristics have been restrained more effectively.

The remainder of this paper is organized as follows. In the Section 2, the piezoelectric actuated stage is modeled by a discrete second order linear equation with KP operators. The design of the adaptive tracking control law is presented in Section 3. The Section 4 performs the experimental setup and experimental results. Followed by the conclusion in Section 5.

## 2. Hysteresis Modeling of the Piezoelectric Actuated Stage

**Figure 1:**
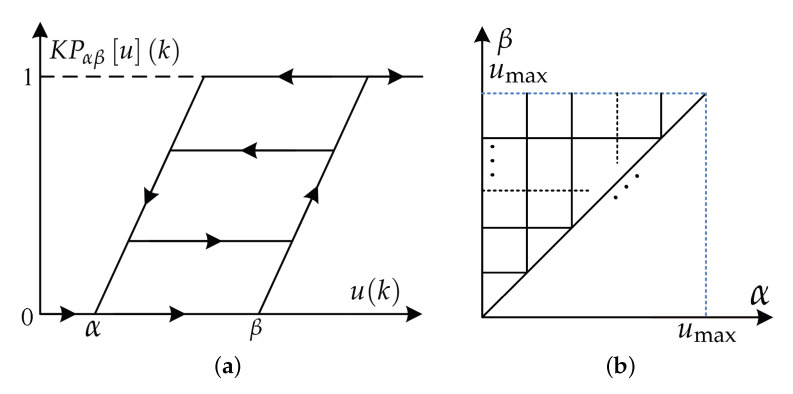
Structure chart of the Krasnosel’skii-Pokrovskii (KP) kernel and Preisach plane. (**a**) KP kernel; (**b**) Preisach plane.

The piezoelectric actuated stage is a complex nonlinear system; it is made up of the mechanical platform and piezoelectric actuator. The mechanical platform is modeled using the mass spring damping system, and the piezoelectric actuator is regarded as an driving force with the voltage input. In general, the dynamic equation of the piezoelectric actuated stage is expressed as

(1) $$\begin{array}{c} m\ddot{y}\left(t\right)+b_{1}\dot{y}\left(t\right)+b_{2}y\left(t\right)=H_{o}\left(t\right), \end{array}$$

where y(t) is the output displacement of the piezoelectric actuated stage, $H_{o}\left(t\right)$ is the driving force from the piezoelectric actuator, and it has severe hysteresis. *m*, $b_{1}$, and $b_{2}$ are the mass, friction coefficient, and stiffness factor of the mechanical platform. In this study, we use a discrete second order linear equation to express the system (1), and its mathematical description is written as:

(2) $$\begin{array}{c} a\left(z^{-1}\right)y\left(k\right)=z^{-2}b\left(z^{-1}\right)H_{o}\left(k\right), \end{array}$$

where the $z^{-1}$ is the delay operator; $b\left(z^{-1}\right)$ is replaced by $b_{0}$, and $b_{0}>0$ is unknown. The $a\left(z^{-1}\right)$ is expressed as:

(3) $$\begin{array}{c} a\left(z^{-1}\right)=1+a_{1}z^{-1}+a_{2}z^{-2}, \end{array}$$

where the parameters $a_{1}$ and $a_{2}$ are unknown. In this paper, we use the KP operator to describe the hysteresis of the piezoelectric actuator, the $H_{o}\left(k\right)$ is replaced by the KP operator formula. So, the whole piezoelectric actuated stage system is expressed as:

(4) $$\begin{array}{c} \left\{\begin{array}{c} a\left(z^{-1}\right)y\left(k\right)=z^{-2}b\left(z^{-1}\right)H\left[u\right]\left(k\right)\;\;\;\;\;\;\;\;\;\;\;\;\;\;\;\;\;\\ H\left[u\right]\left(k\right)={\;\int \int \;}_{\alpha \leq \beta }p\left(\alpha ,\beta \right)KP_{\alpha \beta }\left[u\right]\left(k\right)d\alpha d\beta_{,} \end{array}\right. \end{array}$$

where u(k) is the input of the piezoelectric actuated stage at time *k*, respectively; $KP_{\alpha \beta }\left[u\right]\left(k\right)$ is the output of the elementary KP kernel at time *k*, which is shown in Figure 1a. $H\left[u\right]\left(k\right)$ is the elementary KP kernel with the superimposed density weight ($p\left(\alpha ,\beta \right)$) [19]. $KP_{\alpha \beta }\left[u\right]\left(k\right)$ is expressed as:

(5) $$\begin{array}{c} KP_{\alpha \beta }\left[u\right]\left(k\right)=\left\{\begin{array}{cc} \max\left[\xi \left(k\right),r\left(u(k)-\beta \right)\right] & \mathrm{if}\;u\left(k-1\right)<u\left(k\right)\\ \min\left[\xi \left(k\right),r\left(u(k)-\alpha \right)\right] & \mathrm{if}\;u\left(k-1\right)>u{\left(k\right)}_{,} \end{array}\right. \end{array}$$

where α and β represent the thresholds of the KP operator. As shown in Figure 1a, it influences the width of the hysteresis loop of the KP operator. r(·) is the boundary auxiliary function, which is expressed as:

(6) $$\begin{array}{c} r\left[u\right]\left(k\right)=\left\{\begin{array}{cc} 0 & u\left(k\right)<0\\ u_{\max}*u\left(k\right)/\varrho & 0\leq u(k)\leq \varrho \\ u_{\max} & u\left(k\right)>\varrho_{,} \end{array}\right. \end{array}$$

where ϱ is the slope of the boundary auxiliary function, it is calculated by $\varrho =\left(u_{\max}-u_{\min}\right)/\left(L-1\right)$, $u_{\max}$ and $u_{\min}$ are the maximum and minimum values of the model input. Next, the Preisach plane is divided into a mesh using the *L* for numerically calculating the KP model in Figure 1b. For more descriptions of the Preisach plane, the reader can find it in [19]. ξ(k) is the memory value of the KP operator and it is expressed as:

(7) $$\begin{array}{c} \xi \left(k\right)=\left\{\begin{array}{cc} 0 & \mathrm{for}\;\;k=0\\ KP_{\alpha \beta }\left[u\right]\left(k\right) & \mathrm{for}\;\mathrm{sign}\left(\dot{u}\left(k\right)\right)=-\mathrm{sign}\left(\dot{u}\left(k-1\right)\right)\\ \xi (k-1) & \mathrm{for}\;\mathrm{sign}\left(\dot{u}\left(k\right)\right)=\mathrm{sign}{\left(\dot{u}\left(k-1\right)\right)}_{,} \end{array}\right. \end{array}$$

where sign (·) is the signum function. For ease of the algorithm design, we discrete the KP operator on Preisach plane as follows:

(8) $$\begin{array}{c} H\left[u\right]\left(k\right)=\sum_{i=1}^{N}KP_{\alpha \beta }^{i}\left[u\right]\left(k\right)p_{i}\left(\alpha ,\beta \right), \end{array}$$

where *N* is the number of the KP kernel, it is calculated using N=(L+1)(L+2)/2.

**Figure 2:**
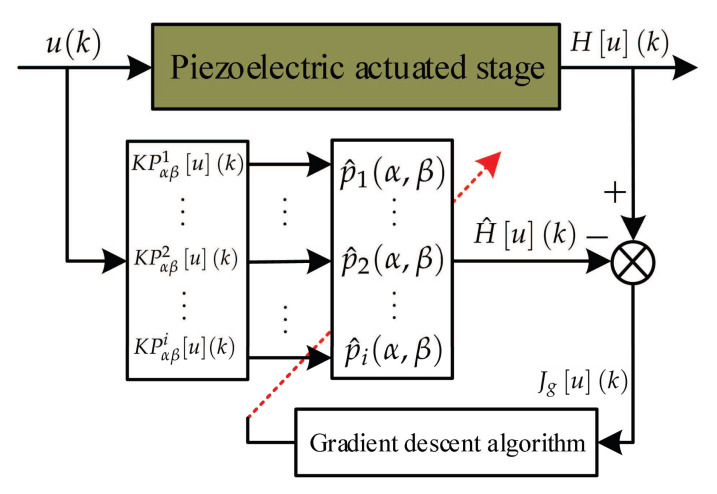
Identification structure diagram of the KP operators with the gradient descent algorithm.

In this study, we use the KP operator to represent the hysteresis of the piezoelectric actuated stage. So, the unknown weights must be obtained at first. Next, we use the gradient descent algorithm to identify the unknown weights of the KP operator. The identification structure diagram of the KP operators are shown in Figure 2. The input signal is used to actuate the KP kernel, then these KP kernels are superimposed with the weights to calculate the output of the KP operators. The output of the KP operator with the estimated weights is expressed as:

(9) $$\begin{array}{c} \hat{H}\left[u\right]\left(k\right)=\sum_{i=1}^{N}KP_{\alpha \beta }^{i}\left[u\right]\left(k\right){\hat{p}}_{i}\left(\alpha ,\beta \right), \end{array}$$

where ${\hat{p}}_{i}\left(\alpha ,\beta \right)$ is the estimated weights. Next, we set the objective function $J_{g}\left(k\right)$ as the sum of the squared modeling error defined by

(10) $$\begin{array}{c} J_{g}\left[u\right]\left(k\right)={\left[H\left[u\right]\left(k\right)-\hat{H}\left[u\right]\left(k\right)\right]}^{2}/2, \end{array}$$

When the value of $J_{g}\left[u\right]\left(k\right)$ is equal or small than default value, the estimated weights ${\hat{p}}_{i}\left(\alpha ,\beta \right)$ are close to the desired values, the output of the KP operators is consistent with the hysteresis output of the piezoelectric actuated stage. Otherwise, it is necessary to calculate $\Delta p_{i}\left(k\right)$ for adjusting the weight values. The adjustment criterion is shown as:

(11) $$\begin{array}{cc} \Delta p_{i}\left(k\right) & =\eta_{g}\frac{\partial J_{g}\left(k\right)}{\partial p_{i}\left(k\right)}\\ \; & =\eta_{g}\left(H\left[u\right]\left(k\right)-\hat{H}\left[u\right]\left(k\right)\right)KP_{\alpha \beta }^{i}\left[u\right]\left(k\right), \end{array}$$

where $\eta_{g}$ is the learning factor, which decides the convergence rate of the gradient descent algorithm. The unknown weights of the KP operator are updated based on the gradient descent algorithm as following:

(12) $$\begin{array}{c} p_{i}\left(k+1\right)=p_{i}\left(k\right)+\Delta p_{i}\left(k\right). \end{array}$$

According to the system model (4), when the input and output of the piezoelectric actuated stage are known, the unknown parameters of the piezoelectric actuated stage model include $a_{0}$, $a_{1}$, $b_{0}$, and p(α,β). In this study, the density weight p(α,β) has been obtained using the gradient descent algorithm by the offline way; other unknown parameters are adaptively acquired using the proposed adaptive control approach.

## 3. Adaptive Tracking Control Law Design

The purpose of this paper is to use the proposed controller to obtained the appropriate input voltage u(k), so that the output y(k) of the piezoelectric actuated stage can track the desired trajectory signal $y_{d}\left(k\right)$ precisely. The adaptive control strategy is a comment control method to make the output of the nonlinear system with unknown parameters track the trajectory signal. In this section, we put forth an adaptive tracking control law based on a new design approach to mitigate the hysteresis of the piezoelectric actuated stage and achieve its high-precision tracking control. At first, the initial error surface is defined as:

(13) $$\begin{array}{c} \sigma \left(k+2\right)=c\left(z^{-1}\right)\left(y\left(k+2\right)-y_{d}\left(k+2\right)\right), \end{array}$$

where $c\left(z^{-1}\right)=1+c_{1}z^{-1}+c_{2}z^{-2}$, and the coefficient $c_{i}$ is chosen such that the $c\left(z^{-1}\right)$ is Hurwitz. It can be shown that, if $\lim_{k\to \infty }\sigma \left(k\right)=0$, $\lim_{k\to \infty }\left(y\left(k\right)-y_{d}\left(k\right)\right)=0$ exponentially. Since the density weights of the KP operators are obtained, we decompose the hysteresis part and the linear part of the system model on the error surface to facilitate the design of adaptive tracking controller, and the new error surface $\sigma \left(k+2\right)$ is

(14) $$\begin{array}{cc} \sigma \left(k+2\right)= & g\left(z^{-1}\right)H\left[u\right]\left(k\right)+f\left(z^{-1}\right)y\left(k\right)-c\left(z^{-1}\right)y_{d}\left(k+2\right), \end{array}$$

where $g\left(z^{-1}\right)=g_{0}+g_{1}z^{-1}$, $f\left(z^{-1}\right)=f_{0}+f_{1}z^{-1}$; and the parameters $g_{0}$, $g_{1}$, $f_{0}$, and $f_{1}$ can uniquely be determined in [32]. Substituting the KP operators (8) into Equation (14), one can express the error surface $\sigma \left(k+2\right)$ as:

(15) $$\begin{array}{cc} \sigma \left(k+2\right)= & \phi^{T}\left(k\right)\theta +g_{0}\sum_{i=1}^{N}KP_{\alpha \beta }^{i}\left[u\right]\left(k\right)p_{i}\left(\alpha ,\beta \right)\\ \; & +g_{1}\sum_{i=1}^{N}KP_{\alpha \beta }^{i}\left[u\right]\left(k-1\right)p_{i}\left(\alpha ,\beta \right)-c\left(z^{-1}\right)y_{d}\left(k+2\right), \end{array}$$

where $\phi \left(k\right)={\left[y(k),y(k-1)\right]}^{T}$, $\theta ={\left[f_{0},f_{1}\right]}^{T}$. For now, we assume that all of the parameters are known. According to Equation (15), we get that the input u(k) satisfies

(16) $$\begin{array}{cc} g_{0}\sum_{i=1}^{N}KP_{\alpha \beta }^{i}\left[u\right]\left(k\right)p_{i}\left(\alpha ,\beta \right)= & -g_{1}\sum_{i=1}^{N}KP_{\alpha \beta }^{i}\left[u\right]\left(k-1\right)p_{i}\left(\alpha ,\beta \right)\\ \; & \;\;\;-\phi^{T}\left(k\right)\theta +c\left(z^{-1}\right)y_{d}\left(k+2\right), \end{array}$$

the error surface σ(k+2)=0, the actual output of the piezoelectric actuated stage is consistent with the desired trajectory signal. Next, we write the estimated values of the parameters as $\hat{g}\left(k\right)={\left[{\hat{g}}_{0}\left(k\right),{\hat{g}}_{1}\left(k\right)\right]}^{T}$, $\hat{\theta }\left(k\right)={\left[{\hat{f}}_{0}\left(k\right),{\hat{f}}_{1}\left(k\right)\right]}^{T}$. The unknown parameters on the right side of the Equation (15) are replaced by the estimated values, we define $\bar{\sigma }\left(k\right)$ as $\bar{\sigma }\left(k\right)=\sigma \left(k\right)+c\left(z^{-1}\right)y_{d}\left(k\right)$. Then, the estimation error is expressed as:

(17) $$\begin{array}{cc} e(k) & =\bar{\sigma }\left(k\right)-\phi^{T}\left(k-2\right)\hat{\theta }\left(k-1\right)-{\hat{g}}_{0}\sum_{i=1}^{N}KP_{\alpha \beta }^{i}\left[u\right]\left(k\right)p_{i}\left(\alpha ,\beta \right)\\ \; & \;\;\;\;\;\;\;\;\;\;\;\;\;\;\;\;\;\;\;\;\;\;\;\;\;\;\;\;\;\;\;\;\;\;\;\;\;\;\;\;\;\;\;\;\;\;\;\;-{\hat{g}}_{1}\sum_{i=1}^{N}KP_{\alpha \beta }^{i}\left[u\right]\left(k-1\right)p_{i}\left(\alpha ,\beta \right), \end{array}$$

Substituting Equation (15) into Equation (17), the estimated error e(k) can be rewritten as:

(18) $$\begin{array}{cc} e(k) & =-y\left(k-2\right)\cdot {\tilde{f}}_{0}\left(k-1\right)-y\left(k-3\right)\cdot {\tilde{f}}_{1}\left(k-1\right)\\ \; & \;\;\;\;\;\;\;-{\tilde{g}}_{0}\sum_{i=1}^{N}KP_{\alpha \beta }^{i}\left[u\right]\left(k\right)p_{i}\left(\alpha ,\beta \right)-{\tilde{g}}_{1}\sum_{i=1}^{N}KP_{\alpha \beta }^{i}\left[u\right]\left(k-1\right)p_{i}\left(\alpha ,\beta \right), \end{array}$$

where the new variables are ${\tilde{f}}_{i}\left(k\right)={\hat{f}}_{i}\left(k\right)-f_{i}\left(k\right),\left(i.e.,\;\tilde{\theta }\left(k\right)\right)$, ${\tilde{g}}_{i}\left(k\right)={\hat{g}}_{i}\left(k\right)-g_{i}\left(k\right)$, (i=0,1). To estimate unknown parameters of the linear part, the cost function is set as:

(19) $$\begin{array}{c} J\left(f_{i},g_{i}\right)\left(k\right)=\frac{1}{2}e^{2}\left(k\right). \end{array}$$

Next, we adopt the following Equation (20) to adjust unknown parameters adaptively.

(20) $$\begin{array}{cc} \dot{\tilde{\theta }}\left(k\right) & =-\gamma \frac{\partial J(k)}{\partial \tilde{\theta }\left(k\right)}=-\gamma e\left(k\right)\frac{\partial e(k)}{\partial \tilde{\theta }\left(k\right)}\\ {\dot{\tilde{g}}}_{i}\left(k\right) & =-\gamma \frac{\partial J(k)}{\partial {\tilde{g}}_{i}\left(k\right)}=-\gamma e\left(k\right){\frac{\partial e(k)}{\partial {\tilde{g}}_{i}\left(k\right)}}_{,} \end{array}$$

where γ is the adjustment speed of the estimated parameters, and it satisfies 0<γ<1. If a fast adaptation of the unknown parameters is needed, the γ should be increased in the range 0<γ<1. Substituting Equation (18) into Equation (20), the adjustment results for each parameter can be derived as follows:

(21) $$\begin{array}{cc} {\hat{f}}_{0}\left(k\right) & ={\hat{f}}_{0}\left(k-1\right)-\gamma \cdot e\left(k\right)\cdot y{\left(k-2\right)}_{,}\\ {\hat{f}}_{1}\left(k\right) & ={\hat{f}}_{1}\left(k-1\right)-\gamma \cdot e\left(k\right)\cdot y{\left(k-3\right)}_{.} \end{array}$$

(22) $$\begin{array}{cc} {\hat{g}}_{0}\left(k\right) & ={\hat{g}}_{0}\left(k-1\right)-\gamma \cdot e\left(k\right)\cdot \sum_{i=1}^{N}KP_{\alpha \beta }^{i}\left[u\right]\left(k\right)p_{i}{\left(\alpha ,\beta \right)}_{,}\\ {\hat{g}}_{1}\left(k\right) & ={\hat{g}}_{1}\left(k-1\right)-\gamma \cdot e\left(k\right)\cdot \sum_{i=1}^{N}KP_{\alpha \beta }^{i}\left[u\right]\left(k-1\right)p_{i}{\left(\alpha ,\beta \right)}_{.} \end{array}$$

The unknown parameters are estimated by Equation (21) and Equation (22). The convergent of the parameters estimated method is not proved in this paper, the more discussions of this method, the reader may refer to [33]. Next, we propose the adaptive control law (23) to eliminate the hysteresis and achieve the high-precision trajectory tracking control for the piezoelectric actuated stage.

(23) $$\begin{array}{cc} u\left(k+1\right)= & u\left(k\right)-\eta \cdot {\left(\psi \left(k\right)-{\hat{g}}_{0}\sum_{i=1}^{N}KP_{\alpha \beta }^{i}\left[u\right]\left(k\right)p_{i}\left(\alpha ,\beta \right)\right)}_{,} \end{array}$$

where the constant η influences the adjustment speed of the input voltage, $\psi \left(k\right)$ is expressed as:

(24) $$\begin{array}{cc} \psi \left(k\right) & =-\phi^{T}\hat{\theta }\left(k\right)-{\hat{g}}_{1}\sum_{i=1}^{N}KP_{\alpha \beta }^{i}\left[u\right]\left(k-1\right)p_{i}\left(\alpha ,\beta \right)\\ \; & \;\;\;\;\;\;\;\;\;\;\;\;\;\;\;\;\;\;\;\;\;\;\;\;\;\;\;\;\;\;\;\;\;+c\left(z^{-1}\right)y_{d}\left(k+2\right)+\kappa \sigma \left(k\right), \end{array}$$

where κ is the weight factor. Based on the Equation (16), if $\psi \left(k\right)$ satisfies the following equation:

(25) $$\begin{array}{c} \psi \left(k\right)=\sum_{i=1}^{N}KP_{\alpha \beta }^{i}\left[u^{*}\right]\left(k\right)p_{i}{\left(\alpha ,\beta \right)}_{,} \end{array}$$

where $u^{*}\left(k\right)$ is equal to the ideal input voltage, and the σ(k) will tend to zero. However, the actual input voltage $u^{*}\left(k\right)$ cannot be accurately calculated. This paper use a weakened condition of Equation (25): it satisfies that $\left|\sum_{i=1}^{N}KP_{\alpha \beta }^{i}\left[u^{*}\right]\left(k\right)p_{i}\left(\alpha ,\beta \right)-\psi \left(k\right)\right|\leq \varepsilon$, for a given admissible error bound ε. So, the adaptive tracking control law (23) can make the tracking error e(k) be approximated to a tolerable error range at the k→∞.

## 4. Simulation and Experimental Results

### 4.1. Simulation Results

**Figure 3:**
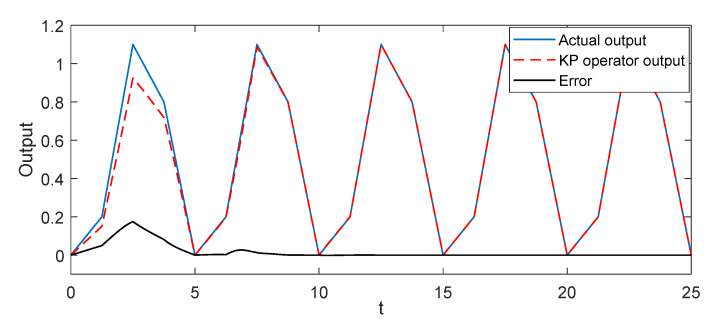
Simulation results of the KP model with the gradient descent algorithm under the triangular wave voltage signal.

**Figure 4:**
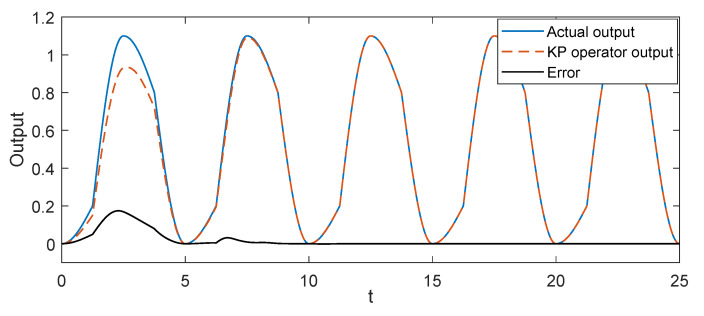
Simulation results of the KP model with the gradient descent algorithm under the sine wave voltage signal.

In this part, we conducted some simulation studies of the KP kernel with the weights to describe the hysteresis in the piezoelectric actuated stage. In simulation, we used three KP kernel with the different weights (which were 0.2, 0.6, and 0.3) as the actual system, then the gradient descent algorithm was used to identify the unknown weights for verifying the validity of the proposed KP operator modeling method. The simulation results are shown in Figure 3 and Figure 4. When the input signal was the triangular wave, it is obvious that the modeling error was close to zero after a period. To avoid the contingently of the simulation results, the sine wave signal was used as the input signal; the output of the KP operators based on the gradient descent algorithm was consistent with the output of actual system after a period from the Figure 4. Simulation results showed that the proposed KP operators based on the gradient descent algorithm could describe the hysteresis of the piezoelectric actuated stage.

### 4.2. Experimental Results

**Figure 5:**
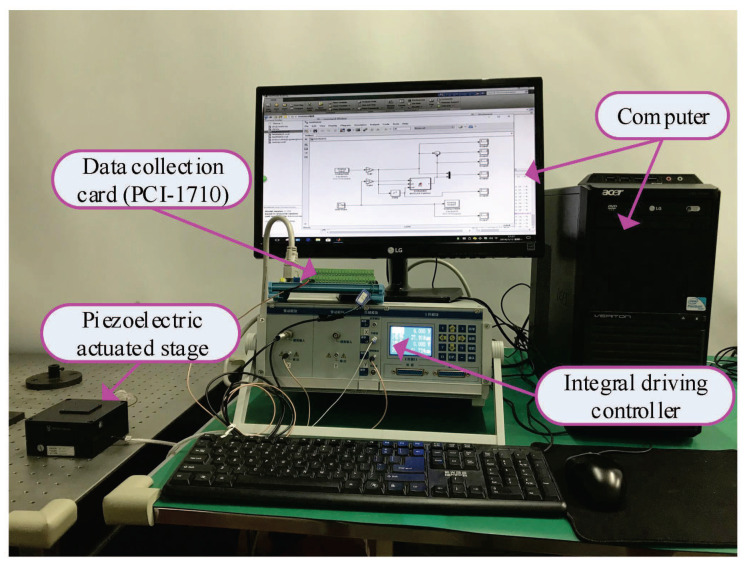
Picture of the piezoelectric actuated stage experimental setup.

In this part, an experimental facility, as shown in Figure 5, was set up to verify the effectiveness of the proposed adaptive tracking control method. It consisted of the piezoelectric actuated stage (MPT-2MRL102A, HuiBo Robotics Technology Co., Ltd, China), the computer, the data collection card (PCI-1710), and the integral driving controller. In the experiment, the control algorithm was implemented using the Matlab/simulink software in the host computer. The integral driving controller with the open-loop mode drove the piezoelectric actuated stage to generate the output displacement. The data collection card was used to handle the D/A or A/D conversion and collect output displacement data of the piezoelectric actuated stage.

**Figure 6:**
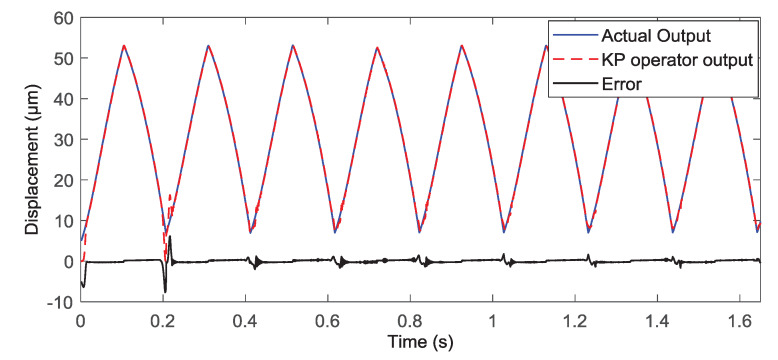
Experimental result of KP operator based on the gradient descent algorithm under the triangular wave voltage signal.

At first, we set triangular wave signal as the driven signal to actuate the piezoelectric actuated stage. The the learning factor $\eta_{g}$ was set as 0.01; the cutting line of the Preisach plane *L* was set as 17 by trial and error in this paper. The identification results are shown in Figure 6. It notes that a larger modeling error was generated as the first period, and then the modeling error decreased gradually to stabilization. The maximum modeling steady error was 0.53 μm, and the corresponding relative error rate was 0.99%. So, the KP operators with the obtained weights could capture the hysteresis characteristics of the piezoelectric actuated stage.

**Figure 7:**
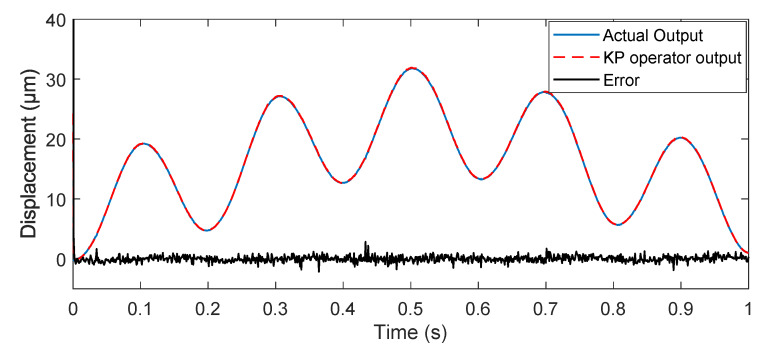
Experimental result of KP operator based on the gradient descent algorithm under the complex wave signal.

To avoid the contingency of modeling experimental results, the complex wave signal (i.e., u=22.5sin(10πt−0.5π)+15sin(2πt−0.5π)+37.5) was the input voltage signal to actuate the piezoelectric actuated stage. The modeling experimental results are shown in Figure 7, the output of the KP operators with the obtained weights were consistent with the actual output of the piezoelectric actuated stage. The maximum modeling steady error rate was 1.21%; it satisfies the requirements of the practical engineering application.

**Figure 8:**
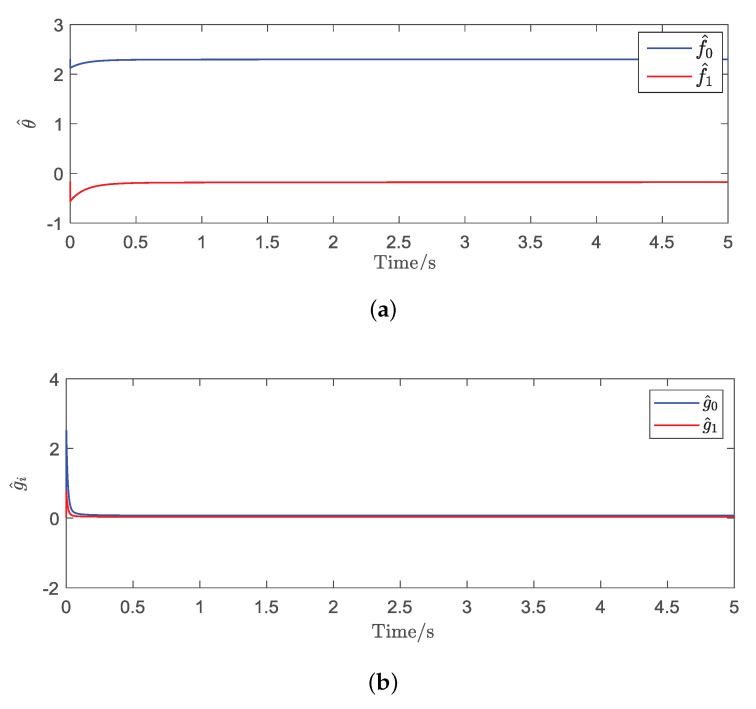
Estimated parameters $\hat{\theta }$ and ${\hat{g}}_{i}$ of the proposed adaptive controller for the triangular wave signal. (**a**) convergent curve of $\hat{\theta }$; (**b**) convergent curve of $\hat{g_{i}}$.

**Figure 9:**
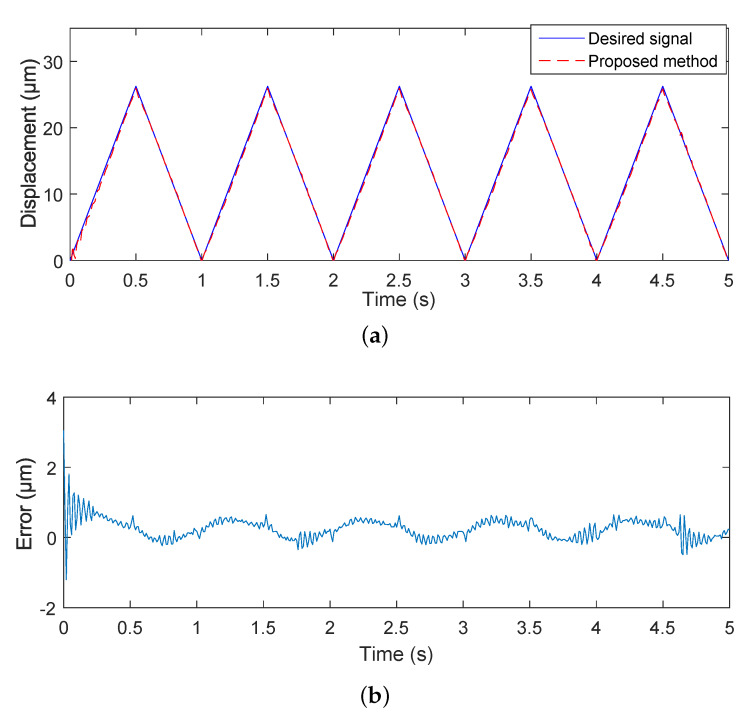
Experimental results of the piezoelectric actuated stage with the triangular wave signal. (**a**) tracking curve of the piezoelectric actuated stage; (**b**) tracking error.

To prove the effectiveness of the proposed adaptive control method, the triangular wave signal was used as the desired trajectory signal to implement the tracking control experiments. The parameters of the proposed controller were set as γ=0.2, η=0.025, and κ=6.8. It can be seen form Figure 8, the estimated unknown parameters could converge to their true values quickly. The convergence time of the proposed adaptive controller was about 0.25 s. Figure 9 shows that the output displacement of the piezoelectric actuated stage based on the proposed adaptive tracking controller was consistent with the desired trajectory signal. The mean tracking error and root-mean-square error of the controller were 0.2526 μm and 0.3063 μm, respectively. The effectiveness of the proposed adaptive controller was verified.

**Figure 10:**
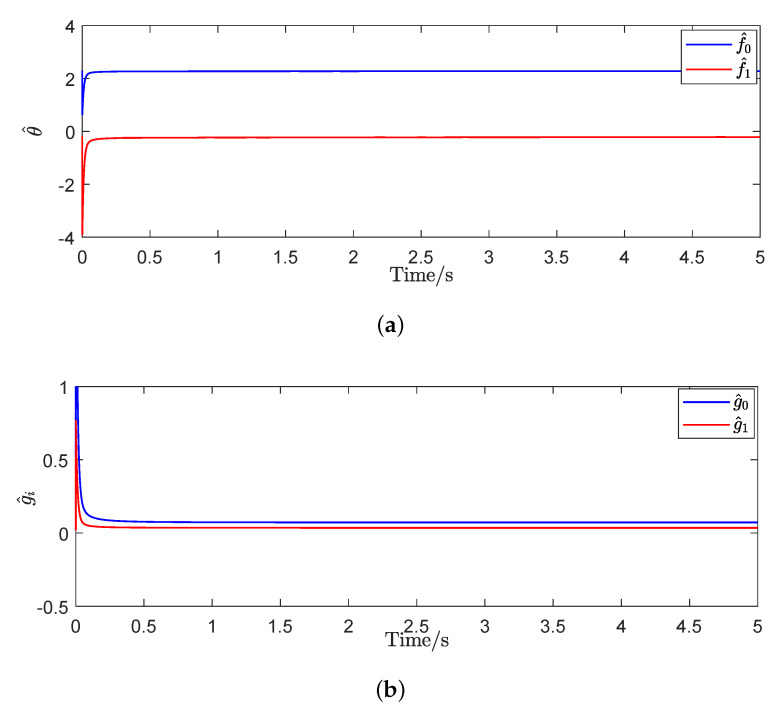
Estimates results of the parameters $\hat{\theta }$ and ${\hat{g}}_{i}$. (**a**) convergent curve of $\hat{\theta }$; (**b**) convergent curve of $\hat{g_{i}}$.

**Figure 11:**
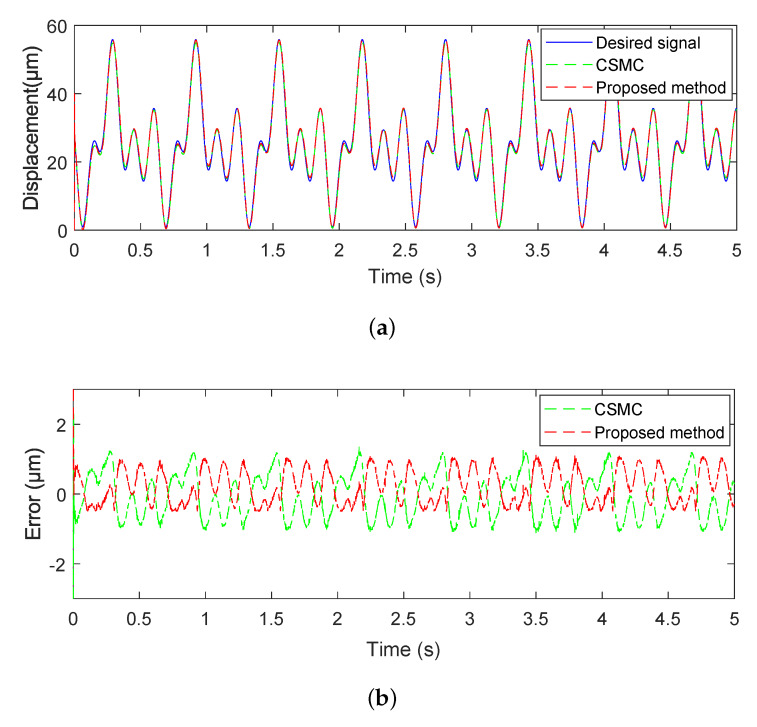
Comparative results of the piezoelectric actuated stage with the complex wave signal. (**a**) tracking curve of the piezoelectric actuated stage; (**b**) tracking error.

Furthermore, in order to further validate the superior performance of the proposed adaptive controller, we chose the complex wave signal as the desired trajectory signal to carry out the comparison tracking experiments. The parameters of the proposed controller were the same as above. The experimental results of the proposed adaptive tracking control method for the piezoelectric actuated stage are shown in Figure 10 and Figure 11. Figure 10 showed that the estimated parameters (${\hat{f}}_{0}$, ${\hat{f}}_{1}$, ${\hat{g}}_{0}$, and ${\hat{g}}_{1})$ could converge quickly to 2.2782, −0.2148, 0.0723, and 0.0355. Figure 11 showed that the proposed adaptive control method achieved more accurate tracking results in comparison with the traditional sliding mode control (SMC) method, where the mean tracking error was reduced by 23.43%. It is concluded from experimental results that the proposed adaptive tracking controller is feasible.

## 5. Conclusions

In this paper, the KP operator with the weights identified by the gradient descent algorithm is used to capture the hysteresis part of the piezoelectric actuated stage. Then, the discrete second order linear equation with the KP operators describes the whole piezoelectric actuated stage system. Next, an adaptive tracking control methodology has been designed for the piezoelectric actuated stage to track the desired trajectory signal. The unknown parameters of the stage systems are adaptively obtained and converge to their desired values. Some comparative tracking experiments are implemented on the piezoelectric actuated stage, where the results verify the superior performance of the proposed adaptive controller in tracking the desired complex wave signal compared with the traditional SMC method.
